# Recombinant elongation factor 1 alpha of *Haemonchus contortus* affects the functions of goat PBMCs

**DOI:** 10.1111/pim.12703

**Published:** 2020-02-28

**Authors:** Muhammad Ehsan, Javaid Ali Gadahi, MingMin Lu, RuoFeng Yan, LiXin Xu, XiaoKai Song, Xing‐Quan Zhu, AiFang Du, Min Hu, XiangRui Li

**Affiliations:** ^1^ MOE Joint International Research Laboratory of Animal Health and Food Safety College of Veterinary Medicine Nanjing Agricultural University Nanjing China; ^2^ State Key Laboratory of Veterinary Etiological Biology Key Laboratory of Veterinary Parasitology of Gansu Province Lanzhou Veterinary Research Institute Chinese Academy of Agricultural Sciences Lanzhou China; ^3^ Department of Veterinary Parasitology Sindh Agriculture University Tandojam Pakistan; ^4^ College of Animal Sciences Zhejiang Provincial Key Laboratory of Preventive Veterinary Medicine Zhejiang University Hangzhou China; ^5^ State Key Laboratory of Agricultural Microbiology College of Veterinary Medicine Huazhong Agricultural University Wuhan China

**Keywords:** elongation factor‐1 alpha, goat, *Haemonchus contortus*, peripheral blood mononuclear cells

## Abstract

Excretory/secretory proteins of *Haemonchus contortus* (HcESPs) intermingle comprehensively with host immune cells and modulate host immune responses. In this study, *H contortus* ES antigen named as elongation factor 1 alpha (HcEF‐1α) was cloned and expressed. The influences of recombinant HcEF‐1α on multiple functions of goat peripheral blood mononuclear cells (PBMCs) were observed in vitro. Immunoblot analysis revealed that rHcEF‐1α was recognized by the serum of goat infected with *H contortus*. Immunofluorescence analysis indicated that rHcEF‐1α was bound on surface of PBMCs. Moreover, the productions of IL‐4, TGF‐β1, IFN‐γ and IL‐17 of cells were significantly modulated by the incubation with rHcEF‐1α. The production of interleukin IL‐10 was decreased. Cell migration, cell proliferation and cell apoptosis were significantly increased; however, nitric oxide production (NO) was significantly decreased. The MHC II molecule expression of cells incubated with rHcEF‐1α was increased significantly, whereas MHC‐I was not changed as compared to the control groups (PBS control and pET32a). These findings indicated that rHcEF‐1α protein might play essential roles in functional regulations of HcESPs on goat PBMC and mediate the immune responses of the host during host‐parasite relationship.

## 1 INTRODUCTION

*Haemonchus contortus* (barber's pole worm) is an important abomasal blood‐sucking nematode parasite of sheep and goat, responsible for economic as well as productive losses. The infection of this nematode induced anaemia, oedema and even death of severely infected animals mainly in warm and humid climate.^1^, ^2^ *H contortus* is the model nematode in the researches of anthelmintic resistance, discovery of effective drug and development of vaccine.^3^, ^4^ Due to the increase of anthelmintic resistance, identifications of new vaccine candidates and therapeutic targets are of great interests among researchers to establish alternative strategies for disease control. During developmental stage in the host, *H contortus* released varieties of molecules termed as excretory/secretory proteins (ESPs), whose functions included suppression or modulation of host immune system for survival of parasite within the host.^5^, ^6^

Elongation factor‐1 alpha (EF‐1α) is a ubiquitous and vastly conserved cytosolic protein among all eukaryotic organisms.^7^ Functionally, EF‐1α is responsible for GTP‐dependent binding of aminoacylated tRNAs to the A site of ribosomes during biosynthesis of protein.^8^ In addition, EF‐1α involved in regulation of wide range of biological processes, such as cell growth and proliferation, vesicular transmission and protein formation, development of mitotic apparatus, signal transduction, DNA replication/repair and apoptosis.^9^, ^10^, ^11^, ^12^, ^13^, ^14^, ^15^ Previous studies reported that EF‐1α proteins were presented in various parasites such as *Trypanosoma brucei*,^16^ *Trichomonas vaginalis*,^17^ *Cryptosporidium hominis*,^18^ *Clonorchis sinensis* and *Brugia malayi*,^19^, ^20^ *Echinococcus granulosus* ^21^ and *Giardia intestinalis*.^22^ Previously, it was suggested that EF‐1α protein allied to the cytoskeleton in apex section of *Cryptosporidium parvum* (*C parvum*), building an important constituent of parasite's invasion apparatus. It was also reported that EF‐1α of *C parvum* played crucial role in mediating host cell entry by the parasite and could be a potential vaccine candidate against *cryptosporidiosis*.^13^ However, in the case of *H contortus*, the biological functions of EF‐1α during parasite invasion and its effects on host immune cells are still a matter of debate.

Previously, in the proteomic analysis of ESPs from *H contortus*, EF‐1α protein was recognized as binding molecule to goat PBMCs at L4, L5 and early adult developmental stages of *H contortus *in vivo.^23^ Meanwhile, the immuno‐suppressant role of HcESPs on goat PBMCs in vitro was also demonstrated.^24^ In recent studies, various binding proteins of *H contortus* were evaluated for their immune‐modulatory properties on goat PBMCs in vitro.^25^, ^26^, ^27^, ^28^, ^29^ However, the immune functional roles of *H contortus* EF‐1α in interaction with host immune cells are still unknown.

In this research, the HcEF‐1α gene was cloned and characterized, and recombinant protein (rHcEF‐1α) was incubated with goat PBMCs to investigate the immune‐regulatory characteristics of rHcEF‐1α on goat PBMCs in vitro, which might be helpful to understand the immune evasion mechanism by the parasite during host‐parasite interactions.

## 2 MATERIAL AND METHODS

### 2.1 Animals, parasite and cells

In this study, local cross‐bred goats about six months to one year old were housed indoor at Nanjing Agricultural University. The anti‐parasitic drug, levamisole (8 mg/kg body weight) was used with two‐week interval to keep goats free from naturally acquired helminths infection. The faecal samples were checked twice per week using standard parasitological techniques, and goats with no sign of helminths infection were used in subsequent experiments. Three biological replicates (three goats), each consisting of three technical replicates (three replicates for each goat), were run for immune and functional studies including immunofluorescence assays, cytokine transcriptional analysis, cell proliferation, nitric oxide production, migration assay, apoptotic activity and MHC molecules expression.

All animals and laboratory experiments were strictly performed in accordance with Animal Ethics Committee, Nanjing Agricultural University, China, and permitted by the science and Technology Agency of Jiangsu Province (ID: SYXK (SU) 2010‐0005). Sprague Dawley (SD) rats with average body weight of 150 g were bought from Experimental Animal Center of Jiangsu, China (Certified: SCXK 2008‐0004).

The *H contortus* strain was maintained by serial passage in helminth‐free goats at laboratory of immunology and molecular parasitology, Nanjing Agricultural University. The adult worms were collected and preserved according to the methods stated previously.^30^ The blood samples were collected from jugular vein of dewormed goats, and PBMCs were isolated by gradient centrifugation standard Ficoll‐hypaque (GE Healthcare) method.^31^ The PBMCs were cultured with the procedure stated previously.^32^

### 2.2 RNA isolation and cDNA synthesis

The total RNA was extracted as previously described.^25^ Briefly, about 100 adult worms were ground in pre‐chilled pestle and mortar with 1‐mL TRIzolfor 30 minutes. After that, 200 μL of trichloromethane was added to the homogenate and centrifuged at 10 000 × g for 15 minutes at 4°C. The supernatant was precipitated by adding 0.25 volume of isopropyl alcohol per each ml of TRIzoland incubated at −20°C for 30 minutes. The RNA was pelleted, washed with 70% ethanol and finally re‐suspended in DEPC‐treated water. The cDNA was synthesized by reverse transcription reaction using cDNA Kit (Takara Biotechnology) according to manufacturer's instructions and preserved at −20°C for downstream applications.

### 2.3 Cloning and expression of HcEF‐1α protein

The open reading frame (ORF) of HcEF‐1α (GenBank/Uniprot: HCOI_00777800/ U6NYV7) was amplified through PCR with specific set of primers. The sense (C**GGATCC**ATGGGCAAAGAAAAGA) and anti‐sense (CCG**CTCGAG**TTATTTCTTCTT‐AGCTCC) primer sequences were added with *BamH* I and *Xho* I restriction sites (bold), respectively. The PCR fragment was inserted into pET32a (+) prokaryotic expression plasmid (TaKaRa) and transformed into competent cells BL21 strain of *E coli* (DE3) in Luria‐Bertani medium (LB) with ampicillin (100 μg/mL). The positive clones were confirmed by sequencing (Invitrogen Biotech Shanghai). Gene characteristics were analysed using BLASTx and translated sequences by BLASTp (http://www.blast.ncbi.nlm.nih.Gov/Blast.cgi). The multiple sequence alignment was done by using Molecular Evolutionary Genetics Analysis 6.1 program (http://www.megasoftware.net/) with protein sequences from different nematode species. The positive clones were cultured in LB media, and protein expression was induced by 1 mmol/L isopropyl‐ß‐D‐thiogalactopyranoside (IPTG; Sigma‐Aldrich, China) at 37°C for 6 hours. The recombinant protein (rHcEF‐1α) was purified by Ni^2+^‐nitrilotriacetic acid (Ni‐NTA) column (GE Healthcare), detected on 12% (w/v) sodium dodecyl sulphate polyacrylamide gel electrophoresis (SDS‐PAGE) and quantified by Bradford method.^33^ The fusion protein of pET32a from the *E coli* BL21 was also produced by the methods described above and used as control protein in multiple functional analyses.

### 2.4 Polyclonal antibodies and western blot analysis of rHcEF‐1α

To produce polyclonal antibody against rHcEF‐1α and fusion vector protein, 0.4 mg of rHcEF‐1α or pET32a was mixed with Freund's complete adjuvant (1:1) and injected into SD subcutaneously. After two weeks, a booster dose of Freund's incomplete (1:1) mixed with the same protein concentration was given by same route followed by three booster doses with 2‐week interval. Finally, 10 days after last immunization, the antisera against rHcEF‐1α or pET32a were collected and stored at −20°C. The serum against *H contortus* (anti‐*H contortus*) was collected from experimentally infected goat,^34^ and sera collected from normal goat and rats were used as negative sera.

Immunoblot analysis was conducted as per earlier described procedure^35^ to detect the specific reactivity of rHcEF‐1α. The protein was electrophoresed on SDS‐PAGE and transferred to polyvinylidene difluoride (PVDF) membrane (Millipore). Then, PVDF strips were allowed to react with anti‐*H contortus* sera or negative rat sera and anti‐pET32a fusion protein sera (1:100 in TBS/0.05% Tween 20) for overnight at 4°C. The strips were further incubated with rabbit anti goat IgG‐HRP (Santa Cruz Biotechnology) as secondary antibody (1:500 dilutions) for 2 hours at 37°C. After each incubation step, the strips were washed with TBST and finally, the bound antibodies were visualized within 3‐5 minutes with 3, 3′‐diaminobenzidine tetrahydrochloride (DAB) kit (Boster Biotech).

### 2.5 Detection of native HcEF‐1α

Parasites were washed in pre‐chilled phosphate‐buffered saline (PBS: Ca^2+^/Mg^2+^‐free; pH 7.4) and disrupted on ice by adding RIPA lysis buffer (Boster Bio). The supernatant was collected by centrifugation at ~10 000 × g for 10 minutes and stored at −80°C. Then, the lysates were electrophoresed on SDS‐PAGE and transferred to the PVDF membrane. HcEF‐1α native protein was detected by Western blot analysis with rat anti‐rHcEF‐1α and goat anti‐rat IgG (Southern, Biotech) as first and second antibodies, respectively, using the same procedure as above.

### 2.6 Detection of rHcEF‐1α binding to goat PBMCs

The isolated PBMCs were incubated with rHcEF‐1α (10 µg/mL) or recombinant pET32a protein (10 µg/mL) and equal volume of PBS as control in a humidified environment (5% CO_2_) at 37°C for 2 hours. Protein binding to the surface of goat PBMCs was confirmed by immunofluorescence assay (IFA).^32^ The PBMCs were settled down on poly‐l‐lysine‐treated glass slides and fixed with 4% paraformaldehyde at room temperature for 20 minutes. After being blocked with 4% BSA in PBS for 1 hour at 37°C, cells were incubated with first antibody rat anti‐rHcEF‐1α‐IgG or rat anti‐pET32a‐IgG (1:100 dilutions) and negative rat IgG (as control) overnight at 4°C followed by goat anti‐rat IgG (Beyotime) as second antibody coupled with Cy3 (1:1000 dilutions) for 30 minutes. The nuclei of corresponding cells were stained with 2‐(4‐Amidinophenyl)‐6‐indolecarbamidine dihydrochloride (DAPI, 1.5 μmol/L; Sigma) for 6 minutes and immersed in Anti‐Fade Fluoromount solution (Beyotime, Biotech). Finally, cells were examined under confocal microscope (LSM710; Zeiss) and images were viewed using the Zeiss software package (Zeiss).

### 2.7 Cytokine productions of PBMCs in response to rHcEF‐1α

The PBMCs at 5 × 10^6^ density were stimulated with ConA (10 μg/mL) and incubated with multiple doses of rHcEF‐1α (10 µg/mL, 20 µg/mL, 40 µg/mL and 80 µg/mL), or pET32a protein (10 and 80 μg/mL) and equal volume of control buffer (PBS) in RPMI 1640 culture medium (containing 100 U/mL penicillin, 100 µg/mL streptomycin, 2 mmol/L l‐glutamine, 10% FBS) at 37°C with 5% CO_2_ for 72 hours. The cells were harvested, and RNA was purified with Total RNA extraction Kit I (OMEGA) followed by cDNA synthesis by using ThermoScript RT and Oligo (dT) 20 primers (Invitrogen) in accordance with kit instructions. The cytokine transcription level for IL‐2, IL‐4, IL‐6, IL‐10, IL‐17, IFN‐γ, TGF‐β1 and endogenous reference gene (β‐actin) was examined using ABI 7500 Real‐Time PCR System (Applied Biosystems) with following cycling conditions: stage 1: initial denaturation at 95°C for 30 seconds; stage 2: amplification at 95°C for 5 seconds and 60°C for 1 minutes; melting curve stage: 60°C–95°C. The primers specific to each cytokines and reference gene are shown in Table 1. Raw cycle thresholds (Ct), obtained from ABI Prism 7500 software version 2.0.6 (Applied Biosystems), were used in the comparative Ct method (2^−ΔΔ^ *^C^* ^t^ method). The data were obtained from triplicates experiment.

**Table 1:** Primer sequences for real‐time PCR

Primer Name	Forward primer（5′→3′）	Reverse primer（5′→3′）	Amplification efficiency (%)^a^	Correlation coefficients (r2)
β‐Actin	CACCACACCTTCTACAAC	TCTGGGTCATCTTCTCAC	95.41	0.9991
IL‐2	CAAACGGTGCACCTACTTCA	AGCTTGAGGTTCTCGGGATT	96.75	0.9985
IL‐4	GTACCAGCCACTTCGTCCAT	GCTGCTGAGATTCCTGTCAA	98.73	0.9994
IL‐6	CGTCGACAAAATCTCTGCAA	TTCCCTCAAACTCGTTCTGG	97.85	0.9981
IL‐10	CCTTGTCGGAAATGATCCAG	AGGGCAGAAAACGATGACAG	98.68	0.9993
IL‐17	TTGTAAAGGCAGGGGTCATC	GGTGGAGCGCTTGTGATAAT	96.68	0.9978
IFN‐γ	GAACGGCAGCTCTGAGAAAC	GGTTAGATTTTGGCGACAGG	98.02	0.9982
TGF‐β1	CATGAACCGGCCCTTCCT	GAAGTCAATGTAGAGCTGACGAACA	98.98	0.9996

^a^ Amplification efficiency (%) = (10‐1/slope −1) × 100.

### 2.8 Expressions of MHC‐I and MHC‐II molecules of goat monocytes

peripheral blood mononuclear cells were poured into flat‐bottom tissue culture plates (Corning) with culture medium RPMI 1640. The nonsticky cells were discarded by multiple washing steps, and the monocytes sticking to the bottom of the plate^36^ were separated and adjusted at 1 × 10^6^ cells/mL density, whereas the purified monocytes were poured into 24‐well culture plates with different protein concentrations (10 µg/mL, 20 µg/mL, 40 µg/mL and 80 µg/mL) or pET32a protein (10 and 80 μg/mL) and equal volume PBS at 37°C for 24 hours. Afterwards, the monocytes were marked with MHC‐I (MCA2189A647; AbDserotec, BioRad) and MHC‐II (MCA2225PE; AbDserotec) monoclonal antibodies and subjected to flow cytometry analysis at FACSCalibur cytometer (BD Biosciences).

### 2.9 Peripheral blood mononuclear cells proliferation

peripheral blood mononuclear cells proliferation assay was conducted as stated previously in accordance with the manufacturer's protocols of cell counting kit‐8 (CCK‐8).^37^ Briefly, PBMCs (1 × 10^6^ cells/mL) were activated with ConA (10 μg/mL) and incubated for 72 hours at 37°C with 5% CO_2_ with multiple concentrations of rHcEF‐1α (10 µg/mL, 20 µg/mL, 40 µg/mL and 80 µg/mL) or pET32a vector protein (10 and 80 μg/mL) and control buffer (PBS). Four hours before collection of cells, 10 µL of reagent (Beyotime Biotechnology) was added to each well and absorbance was measured in microplate reader (Thermo Scientific) at 450 nm (OD_450_). Data were obtained from three independent experiments.

### 2.10 Cell migration assay

The Millicell^®^ insert with 8.0μm pores (Merck‐Millipore) was used to determine migration activity of goat PBMCs as described earlier.^29^ The 200 μL cells (1 × 10^6^ cells/mL) containing different concentrations of rHcEF‐1α (10 µg/mL, 20 µg/mL, 40 µg/mL and 80 µg/mL) or pET32a protein (10 and 80 μg/mL) and control buffer (PBS) were seeded into the upper chamber, and subsequently, 1300 μL cell culture medium was filled in the lower chamber for 2 hours incubation at 37°C and 5% CO_2_. After that, columns were removed and PBMC passed through polycarbonate layer were figured out by a Neubauer counting chamber. Data were presented as percentage of seeded PBMCs.

### 2.11 Nitric oxide production assay

The goat PBMCs were separated and washed with Ca^2+^/Mg^2+^‐free PBS (pH 7.4). The 100 µL of cells (1 × 10^6^ cells/mL) in DMEM medium were poured in 96‐well plate with varying concentrations of rHcEF‐1α (10 µg/mL, 20 µg/mL, 40 µg/mL and 80 µg/mL) or pET32a protein (10 and 80 μg/mL) and control buffer (PBS). According to the Total Nitric Oxide Assay Kit (Beyotime Biotechnology), intracellular nitrite in PBMCs was measured by using Griess assay.^38^ Absorbance values of the coloured solution were measured using a plate reader (Thermo Scientific) at 540 nm (OD_540_). Data were obtained from three independent experiments.

### 2.12 Cell apoptosis assay

Flow cytometer analysis was carried out as described earlier.^39^ PBMCs were cultured with different concentration of rHcEF‐1α and pET32a protein for 24 hours. The cells were then washed twice with Ca^2+^/Mg^2+^‐free PBS pH 7.4 and were re‐suspended in binding buffer, and apoptosis assay was performed according to the manufacturer's instructions of the Annexin V‐FITC kit (Biovision). Annexin V‐FITC was added to the cell suspension for 15 minutes in the dark at room temperature. The stained cells were analysed by flow cytometry (BD, FACSCalibur) just after addition of propidium iodide (PI, Sigma‐Aldrich) to the cell suspension.

### 2.13 Data analysis

The statistical analyses were performed by using the GraphPad Premier 6.0 software package (GraphPad Prism). Results were presented as mean ± SEM. Comparisons among groups were carried out with one‐way analysis of variance (ANOVA), followed by a Tukey test and considered statistically significant at *P* < .05.

## 3 RESULTS

### 3.1 Cloning and sequence analysis of HcEF‐1α gene

The RT‐PCR generated amplicon of HcEF‐1α gene successfully cloned into pET32a expression vector, and an insert of 1395 bp was verified by enzymatic digestion. The BLASTx and BLASTp bioinformatics tools showed that ORF of HcEF‐1α contained 1395 bp, encoded 464 amino acids and calculated pI of 8.95. Multiple sequence alignment of HcEF‐1α with available sequences of EF‐1α on NCBI database revealed that HcEF‐1α was closely related to the *H contortus* (99%), *Necator americanus* (98%), *Caenorhabditis elegans* (94%), *Ascaris suum* (94%), *Toxocara canis* (94%), *Strongyloides* ratti (92%), *Homo sapiens* (85%) and *Ovis aries* (85%). The predicted polypeptide sequence analysis revealed the presences of characteristic regions in all sequences, including GDP/GTP conversion, GTP hydrolysis and GTP‐induced conformational change. The characteristic sequence components, GHVDSGK (G^14^‐K^20^), DAPG (D^91^‐G^94^), NKMD (N^153^‐ D^156^) and SGX (G^194^‐X^196^) were recognized in the GTP‐binding site of the *H contortus* polypeptides. Moreover, the predicted amino acid sequences identified a conserved motif as a GTP‐binding elongation factor signature “DKLKAERERGITIDI(A/S)” at positions 61 to 76 in all sequences of EF‐1α (Appendix S1). No signal peptide (Appendix S2) or transmembrane domains (Appendix S3) were detected within the protein structure, although 14 T cell and 21 B cell epitopes were predicted in target structure (Appendix S4).

### 3.2 Purification of rHcEF‐1α protein

The IPTG‐induced recombinant HcEF‐1α protein product was rich in supernatant of sonicated bacterial culture. The purified protein had a single band of about 68 kDa rather than the calculated mass of 50 kDa. The larger protein size was due to the extra fused vector protein of 18 kDa (result not shown).

### 3.3 Immunoblot analysis for recombinant and native protein of HcEF‐1α

In Western blot analysis, rHcEF‐1α was detected with serum from goat infected with *H contortus* parasite, but could not be detected by the serum of normal goat. Furthermore, the total soluble proteins of *H contortus* were detected with serum from goat infected with *H contortus* parasite, and the specificity of native EF‐1α protein in whole soluble extracts of *H contortus* could be distinguished by antibodies generated in SD rats immunized with rHcEF‐1α which identified a single band of about 50 kDa, but could not be recognized with the sera from normal rats (result not shown).

### 3.4 Binding of rHcEF‐1α to the surface of goat PBMCs

**Figure 1:**
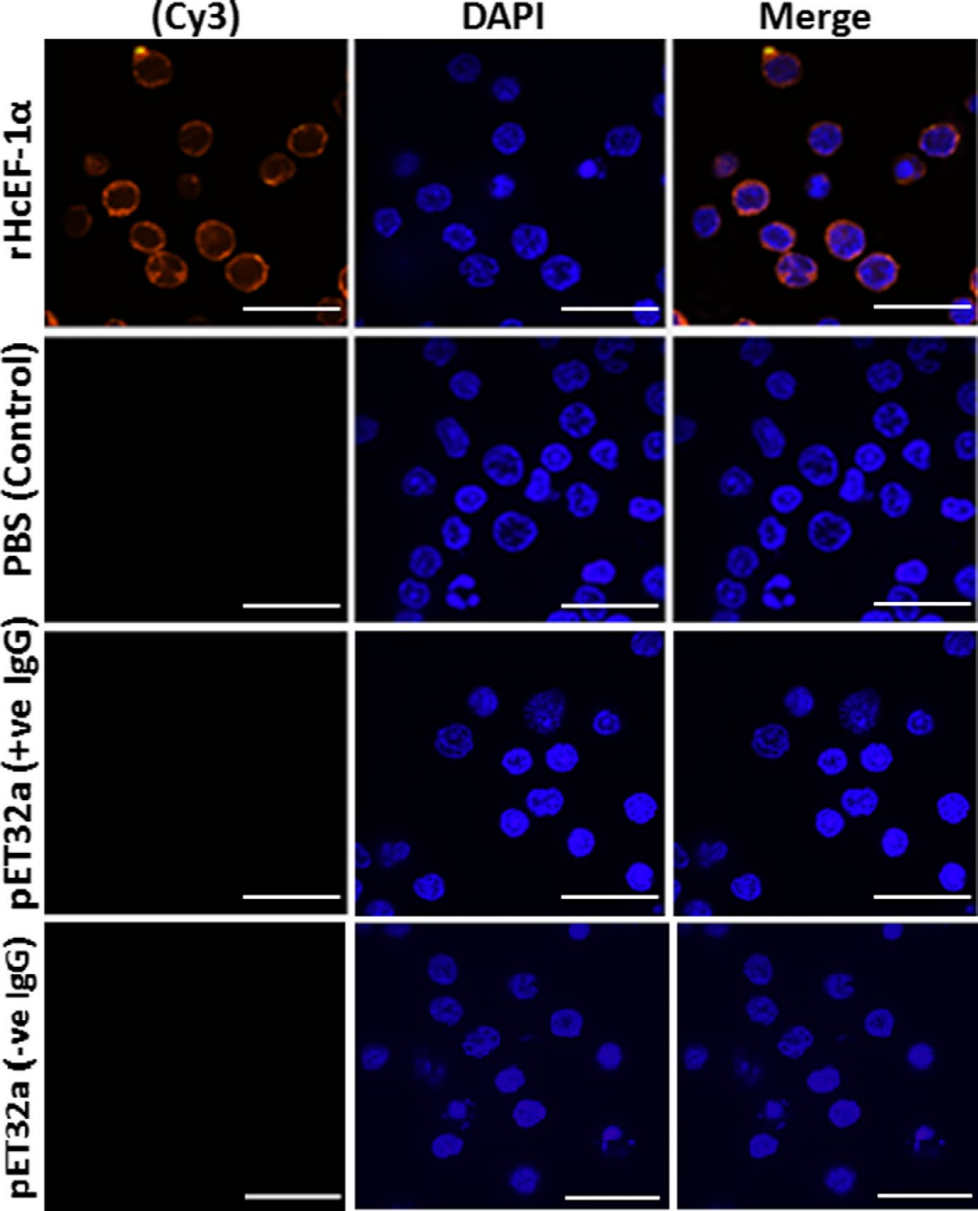
Binding of rHcEF‐1α protein to the goat peripheral blood mononuclear cells (PBMC). Localization was conducted by incubation of PBMC with rat anti‐rHcEF‐1α IgG or rat anti‐pET32a‐IgG and negative rat IgG (as control). Staining of the target protein (red) was detected by the Cy3‐conjugated secondary antibody. Nuclei of corresponding cells were stained with DAPI (blue) and merge corresponding to overlaps of red and blue channels visualized by confocal microscopy. pET32a (+ve IgG): cells pre‐treated with pET32a protein were incubated with rat anti‐pET32a protein IgG. pET32a (‐ve IgG): cells pre‐treated with pET32a protein were incubated with negative rat IgG. No red fluorescence was observed either in PBS control or pET32a control groups. Scale‐bars: 10 µm

Binding of rHcEF‐1α to the surface of goat PBMCs was detected by IFA. As shown in Figure 1, the cultured PBMCs exposed to the first antibody and the secondary antibody coupled with Cy3 strongly indicated binding of target protein (red colour) to the surface of cells. However, no protein binding was observed in control sections, which indicated that only target protein rHcEF‐1α could bind on the surface of PBMC.

### 3.5 Cytokine production levels of PBMCs in response to rHcEF‐1α

**Figure 2:**
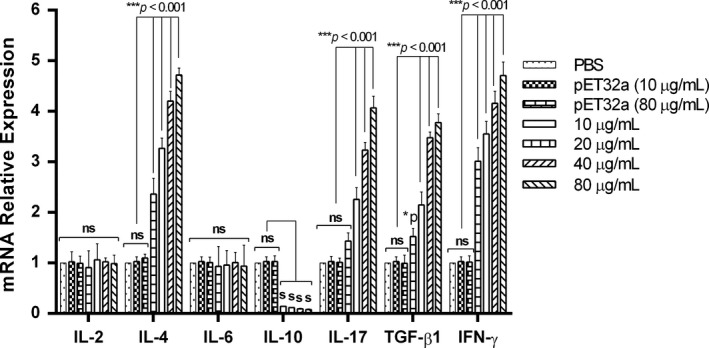
Effects on multiple cytokine mRNA expression in goat peripheral blood mononuclear cells (PBMC) incubated with rHcEF‐1α protein. Cytokine production for IL‐2, IL‐4, IL6, IL‐10, IL‐17, IFN‐γ and TGF‐β1 in the sediments of cell culture was quantified by real‐time PCR. PBMC used for all replicates of distinct treatments in each experimental repetition were derived from the same goat. The data are representative of independent experiments triplicate in each and set as significant at *P < .05, ***P < .001 and letter “a” indicates nonsignificant difference

The PBMCs incubated with rHcEF‐1α showed increased levels of IL‐4, IL‐17, TGF‐β1 and IFN‐γ cytokines (*P* < .001). The level of IL‐10 cytokine was very minute but decreased (*P* < .002). However, the secretion of IL‐2 (*P* < .958) and IL‐6 (*P* < .638) cytokines were not significantly changed to that of pET32a and PBS control groups (Figure 2). Our results indicated that the pET32a fusion protein preparation had no or little immunogenicity at the highest equivalent concentration of rHcEF‐1α (80 µg/mL). Thus, the presence of fusion protein in the rHcEF‐1α did not impact the specificity of the antibody against the rHcEF‐1α or weakly influence it.

### 3.6 PBMC proliferation assay

**Figure 3:**
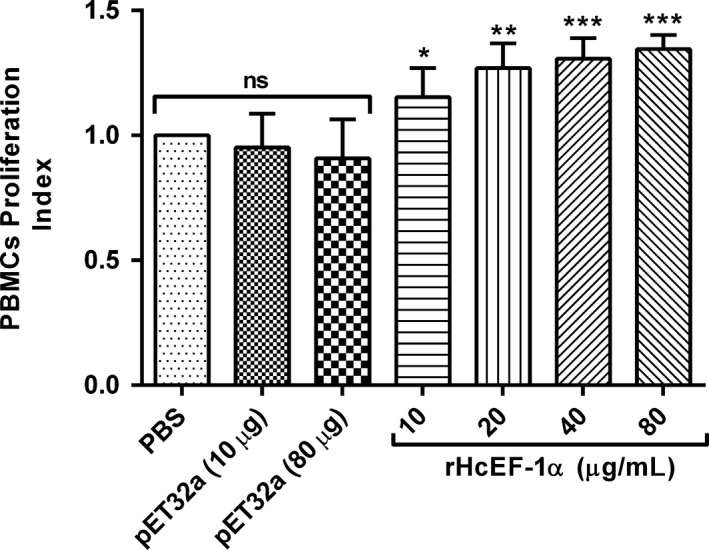
rHcEF‐1α modulated the proliferation of goat peripheral blood mononuclear cells (PBMC) in vitro. Cells were treated with control buffer, pET32a protein and serial concentrations of rHcEF‐1α at 37°C and 5% CO_2_. Proliferation test was conducted by CCK‐8 incorporation after 72 h. Cell proliferation index was calculated considering the OD450 values in controls as 100%. PBMC used for all replicates of distinct treatments in each experimental repetition were derived from the same goat. The data are representative of triplicate experiments (*P < .05, **P < .01 and ***P < .001)

As demonstrated by cell counting kit‐8 (CCK‐8), rHcEF‐1α protein significantly increased the PBMC proliferation at the concentration of 10 µg/mL (*P* < .022), 20 µg/mL (*P* < .002), 40 µg/mL (*P* < .001) and 80 µg/mL (*P* < .001) in contrast to control groups. No significant proliferation was induced between PBS control and pET32a protein groups. Moreover, there was no effect of the pET32a fusion protein preparation at the highest equivalent concentration of rHcEF‐1α (80 µg/mL) (Figure 3).

### 3.7 PBMC migration assay

**Figure 4:**
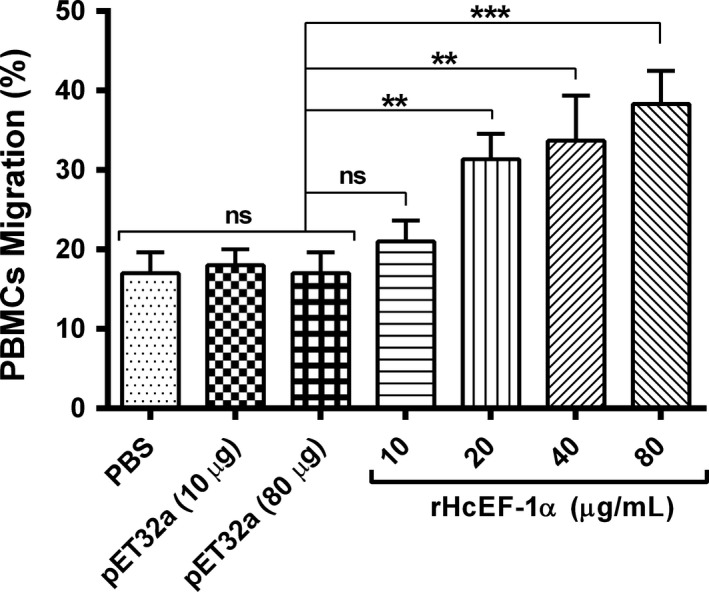
rHcEF‐1α protein promoted the migration of goat peripheral blood mononuclear cells (PBMC). Cells were treated with control buffer, pET32a protein and serial concentrations of rHcEF‐1α. PBMC used for all replicates of distinct treatments in each experimental repetition were derived from the same goat. Data shown here are representative of three independent experiments (**P < .01, ***P < .001, ns nonsignificant)

As shown in Figure 4, compared with the PBS group and pET32a protein groups, the treatment of PBMCs with rHcEF‐1α significantly increased the migration at concentration of 20 µg/mL (31.33 ± 1.856%, *P* < .010), 40 µg/mL (33.67 ± 3.283%, *P* < .029) and 80 µg/mL (38.33 ± 2.404%, *P* < .001). However, there was no difference in percentage of migrated PBMCs at 10 µg/mL protein concentration (21.00 ± 1.528%, *P* < .196) to that of PBS control and pET32a groups (Figure 4). In addition, no significant change of fusion protein preparation was observed at the highest equivalent concentration of rHcEF‐1α (80 µg/mL).

### 3.8 Measurement of nitric oxide production

**Figure 5:**
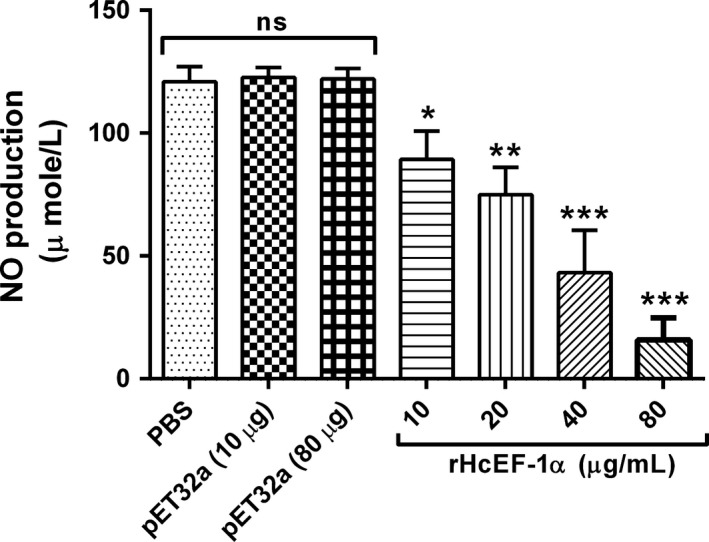
rHcEF‐1α decreased the intracellular nitric oxide production in goat peripheral blood mononuclear cells (PBMC). Cells were treated with control buffer, pET32a protein and differential concentration of rHcEF‐1α at 37°C and 5% CO_2_. The NO concentration in the PBMCs was measured by Griess assay. PBMC used for all replicates of distinct treatments in each experimental repetition were derived from the same goat. The data are presented as the mean ± SEM and representative of triplicate experiments (*P < .05, **P < .01, ***P < .001, ns, nonsignificant)

The intracellular nitrite production in PBMCs was measured by using Griess assay. When PBMCs were incubated with rHcEF‐1α, NO production was gradually decreased at 10 µg/mL (89.26 ± 6.667, *P* < .014), 20 µg/mL (74.98 ± 6.400, *P* < .001), 40 µg/mL (43.15 ± 9.948, *P* < .001) and 80 µg/mL (15.87 ± 5.170, *P* < .001) at dose‐dependent manner (Figure 5) compared to that of PBS control group and pET32a groups. However, no momentous change of pET32a fusion protein was observed at highest equivalent concentration of rHcEF‐1α (80 µg/mL).

### 3.9 rHcEF‐1α induced the apoptosis of PBMCs

**Figure 6:**
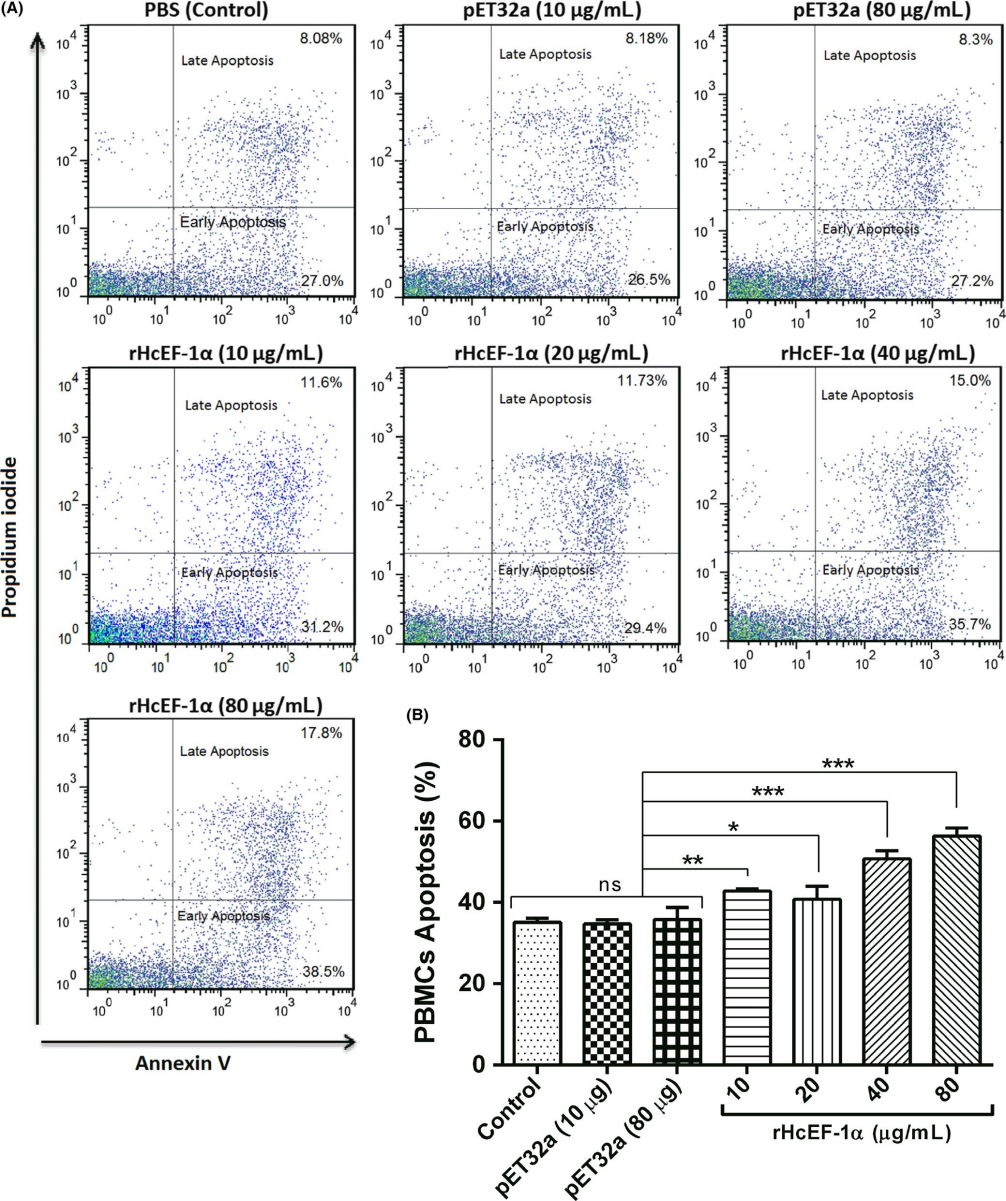
rHcEF‐1α increased apoptosis of goat peripheral blood mononuclear cells (PBMC) at dose‐dependent manner. A, Apoptosis of PBMC were determined by staining with annexin V and PI followed by flow cytometry. The percentages of cells with different staining patterns are shown. B, rHcEF‐1α up‐regulated the apoptosis of goat PBMC at different protein concentrations. PBMC used for all replicates of distinct treatments in each experimental repetition were derived from the same goat. Data are presented as the mean ± SEM (n = 3) of triplicate experiments, an asterisk indicates treatment groups differ significantly at *P < .05, **P < .01 and ***P < .001 from the control

The PBMCs were incubated at various concentrations of rHcEF‐1α (10, 20, 40 and 80 µg/mL) or pET32a protein (10 and 80 μg/mL) and control buffer (PBS), and apoptotic cells were detected by annexin V staining. The results showed that rHcEF‐1α significantly induced apoptosis of PBMCs (*P* < .001) (Figure 6). No significant differences of apoptosis were found between PBS control (35.08 ± 0.5774) and 10 µg/mL of pET32a group (34.68 ± 0.5774). However, the percentage of apoptotic cells was simultaneously increased at 10 µg/mL (42.80 ± 0.2887%), 20 µg/mL (40.73 ± 1.848%), 40 µg/mL (50.70 ± 1.155%) and 80 µg/mL (56.30 ± 1.155%). In addition, there was no significant influence of pET32a protein preparation at highest equivalent concentration of rHcEF‐1α (80 µg/mL) (Figure 6).

### 3.10 rHcEF‐1α increased expression of MHC class II but not MHC‐I of goat monocytes

**Figure 7:**
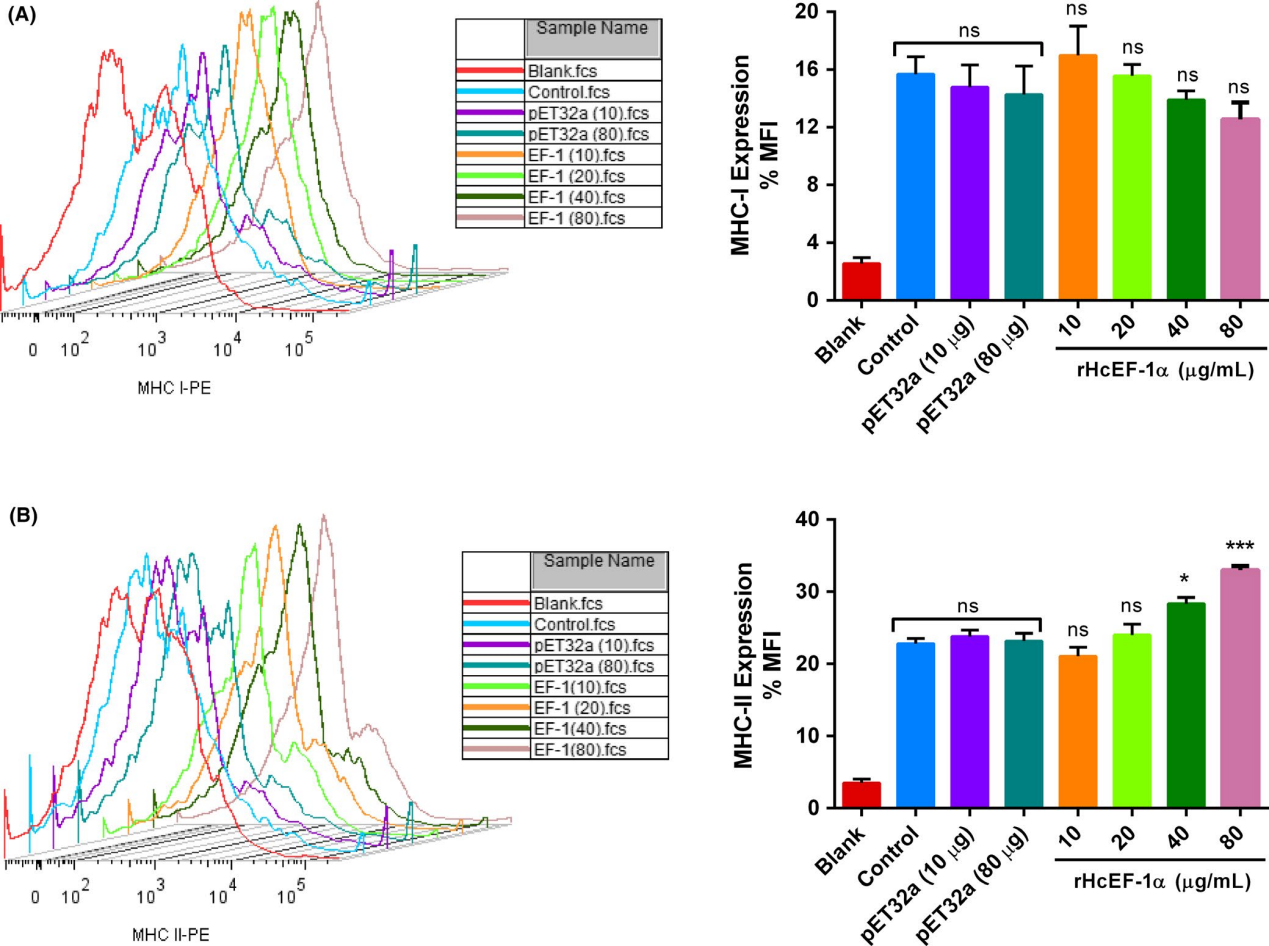
rHcEF‐1α mediated differentially MHC level on goat peripheral blood mononuclear cells (PBMC). Cells were cultured in the presence of control buffer, HisTag protein (pET32a) and multiple concentrations of rHcEF‐1α for 24 h. MHC‐I and MHC‐II expressions were measured by flow cytometric analysis and calculated as the percentage of mean fluorescence intensity (MFI). (A + B, Panel‐1) Stagger offset showing expression level of MHC on monocytes and (A + B, Panel‐2) Bar graphs represent the MFI ± SD of controls. PBMC used for all replicates of distinct treatments in each experimental repetition were derived from the same goat. The data are representative of three independent experiments (*P < .05, ***P < .001 and ns nonsignificant)

As illustrated in Figure 7, the percentage of MHC‐II^+^ cells was increased with 40 µg/mL (28.33 ± 0.8819; *P* < .089) and 80 µg/mL (33.00 ± 0.5774; *P* < .001) rHcEF‐1α compared to that of PBS and pET32a control groups (Figure 7B). However, no significant MHC‐I expressions were noticed (*P* < .967) in the PBMC incubated with differential protein concentrations of rHcEF‐1α (Figure 7A). In addition, there was no significant influence of pET32a protein preparation at highest equivalent concentration of rHcEF‐1α (80 µg/mL).

## 4 DISCUSSION

The EF‐1α is one of the important constituent of *H contortus* excretory and secretory proteins, which play essential role in mediating cellular and humoral immune responses.^40^, ^41^ However, no information is available regarding characterization of *H contortus* EF‐1α. EF‐1α is a profusely expressed and highly conservative cytosolic protein with an approximate molecular mass of 50 kDa,^42^ which contains three domains responsible for GTP binding (Domain I), recognizing the tRNA (Domain II) and attachment of aminoacyl‐tRNA to the ribosome (Domain III).^43^ In this study, sequence analysis of rHcEF‐1α predicted polypeptides revealed the occurrence of GTP‐binding motifs^44^, ^45^ with the representative sequence residues G^14^‐K^20^, D^91^‐G^94^ and N^153^‐ D^156^. These structural similarities within EF‐1α of different species suggested that HcEF‐1α held an essential position at the parasitic cytoskeleton and might play vital roles in regulations of cytoskeletal dynamics by cross‐linkage of actin filaments and microtubules.^46^ In this research, the molecular weight of the native HcEF‐1α was about 50 kDa in somatic extracts of *H contortus* parasite detected by the antibodies of rat against rHcEF‐1α and the molecular weight of rHcEF‐1α was about 68 kDa. Apart from the tag peptides of 18 kDa of the vector, the size of the rHcEF‐1α was identical to that of the native one. Western blot analysis showed that rHcEF‐1α could be recognized by the sera of goat experimentally infected with *H contortus*. The anti‐rHcEF‐1α antibodies used to probe the whole soluble proteins of *H contortus* produced only single band, which means these antibodies did not show cross‐reactivity with other proteins in this parasite. Our results suggested that these antibodies had only specificity against EF‐1α protein of *H contortus*. It indicated that the native HcEF‐1α could enter into the circulatory system of the goat and be recognized by the host immune system.

Parasite ESPs contained many molecules which could modulate or suppress the host immune functions. This interactions was directed by binding of these ESP molecules in the form of receptor‐ligand complexes to the receptors on the surface of the host cells.^6^ Previously, EF‐1α was identified as a binding protein for β‐actin mRNA and filamentous (F)‐actin in cell protrusions,^47^ which suggested that EF‐1α played roles in the regulation of cytoskeletal dynamics to form a partial signalling complex that regulated cell motility and growth via association with the cytoskeleton. In this study, the IFA confirmed that rHcEF‐1α could bind to the surface of goat PBMCs in vitro, which indicated that it might play some regulatory roles on cells during host‐parasite interactions.

During the infection of *H contortus*, the main humoral immune response was generally considered as the main protective response.^48^ IL‐4 was produced by Th2 cells and played key regulation function on the humoral responses. IFN‐γ was the typical cytokine of Th1 cells and mainly regulated the cellular response. However, IL‐10 and TGF‐β1 were produced by Treg cells and usually inhibited the immune responses.^49^, ^50^ It was reported that EF‐2 of *Leishmania donovani* down‐regulated the IL‐10 production in PBMCs form infected patients and in LPS stimulated PBMCs.^51^ Our results suggested that significant increase in IL‐4 and IFN‐γ productions and inhibited level of IL‐10 might enhance the protective immune response against the infection of *H contortus*. However, rHcEF‐1α also increased the production of TGF‐β1. The mechanisms under this contrary effect on both IL‐10 and TGF‐β1 need to be further studied.

The pro‐inflammatory cytokine IL‐17 was secreted by the Th17 cells and related to pathogenesis of numerous helminths parasite.^52^, ^53^, ^54^ A previous study demonstrated that HcESPs were capable to induce IL‐17 cytokine production in PBMCs.^24^ In this research, the results demonstrated that rHcEF‐1α could significantly promote IL‐17 production. It suggested that HcEF‐1α might involve in inflammatory reactions and the pathogenesis of *H contortus*.

Generally, major histocompatibility complex (MHC) class II molecule was constitutively expressed on the surface of APCs and tightly linked with parasitic infection to elicit antibody production and initiation of adaptive immune response.^55^, ^56^ In previous study, it was stated that EF‐1α could play important role to inhibit the parasites by presenting antigen peptides to CD4^+^ T cells and resulted in strong adaptive immune responses through increased level of MHC‐II molecule expression.^57^ In this study, an increased expression level of MHC‐II molecule on monocytes was noted after treatment with rEF‐1α protein. It also indicated that HcEF‐1α could promote the protective immune response against the infection of *H contortus*, identical to the increased IL‐4 level.

Nitric oxide (NO) was released by various cell types mainly monocytes/macrophages that were activated by IFN‐γ and exhibited numerous immune‐regulatory and cytotoxic activities in the immune system.^58^ In the current study, rHcEF‐1α significantly increased the production of IFN‐γ and significantly decreased the NO production at dose‐dependent manner. Previously, it was also documented that rHcFTT‐2 up‐regulated the IFN‐γ production and down‐regulated NO production by goat PBMCs in vitro.^25^ These results suggested that the effects of ESP molecules of *H contortus* on host immune cells were complex, and the real mechanisms under that should be further researched.

Cell proliferation and migration are crucial for generation of immune responses during helminths infection by raising the quantity of effector cells (eosinophils/lymphocytes) and recruiting the effector cells at the infected site.^24^, ^59^ Recently, it was showed that *H contortus* ES‐binding protein (rMiro‐1) increased the cell proliferation as well as percentage of migrated PBMC in vitro.^60^ The present research indicated that rHcEF‐1α significantly increased the proliferation and migration of goat PBMCs. It suggested that this protein played roles in the generation and recruitment of the immune effector cells.

Apoptosis is a complex cellular mechanism occurring in all multicellular organisms and considered as immune‐regulator of host immune responses induced by parasites.^61^ It was reported that increased level of EF‐1α enhanced the apoptosis of cells.^62^ In this research, HcEF‐1α significantly increased the goat PBMC apoptosis at dose‐dependent manner, consistent with the previous research.

In conclusion, our study revealed that the interaction of rHcEF‐1α could enhance goat PBMC to express cytokines IL‐4, IL‐17, IFN‐γ, TGF‐β1 and MHC‐II molecule along with increased levels of cell migration, proliferation and apoptosis. However, rHcEF‐1α incubated with goat PBMC down‐regulated the interleukin IL‐10 and nitric oxide production (NO). These results indicated that this protein might potentially up‐regulate the immune responses during the infection of *H contortus* and might be a candidate for the development of vaccine against the infection of this nematode.

## CONFLICT OF INTEREST

On behalf of all authors, the corresponding author states that there is no conflict of interest.

## AUTHOR CONTRIBUTIONS

XRL designed the project and participated in the coordination and management during the study period. ME performed the experiments and wrote the manuscript. JAG and MML helped in blood sampling, PBMCs isolation and also provided inputs in computational analysis. RFY, XKS and LXX provided new analytical reagents and tools. XQZ, AFD and MH provided necessary amendments in manuscript. All authors read and approved the final version of manuscript.

## Supporting information

Additional fileS1

Additional fileS2

Additional fileS3

Additional fileS4

## Data Availability

The data sets supporting the conclusions of this article are included within the article and its Appendix [Link], [Link], [Link], [Link].
