# Supplementary material for: Recombinant elongation factor 1 alpha of Haemonchus contortus affects the functions of goat PBMCs
Source: Parasite Immunol. 2020 Feb 28;42(5):e12703. doi: 10.1111/pim.12703 (PMC7187238; doi:10.1111/pim.12703)
Supplement: Supplementary file 1 — Additional fileS1 [file PIM-42-e12703-s001.docx]

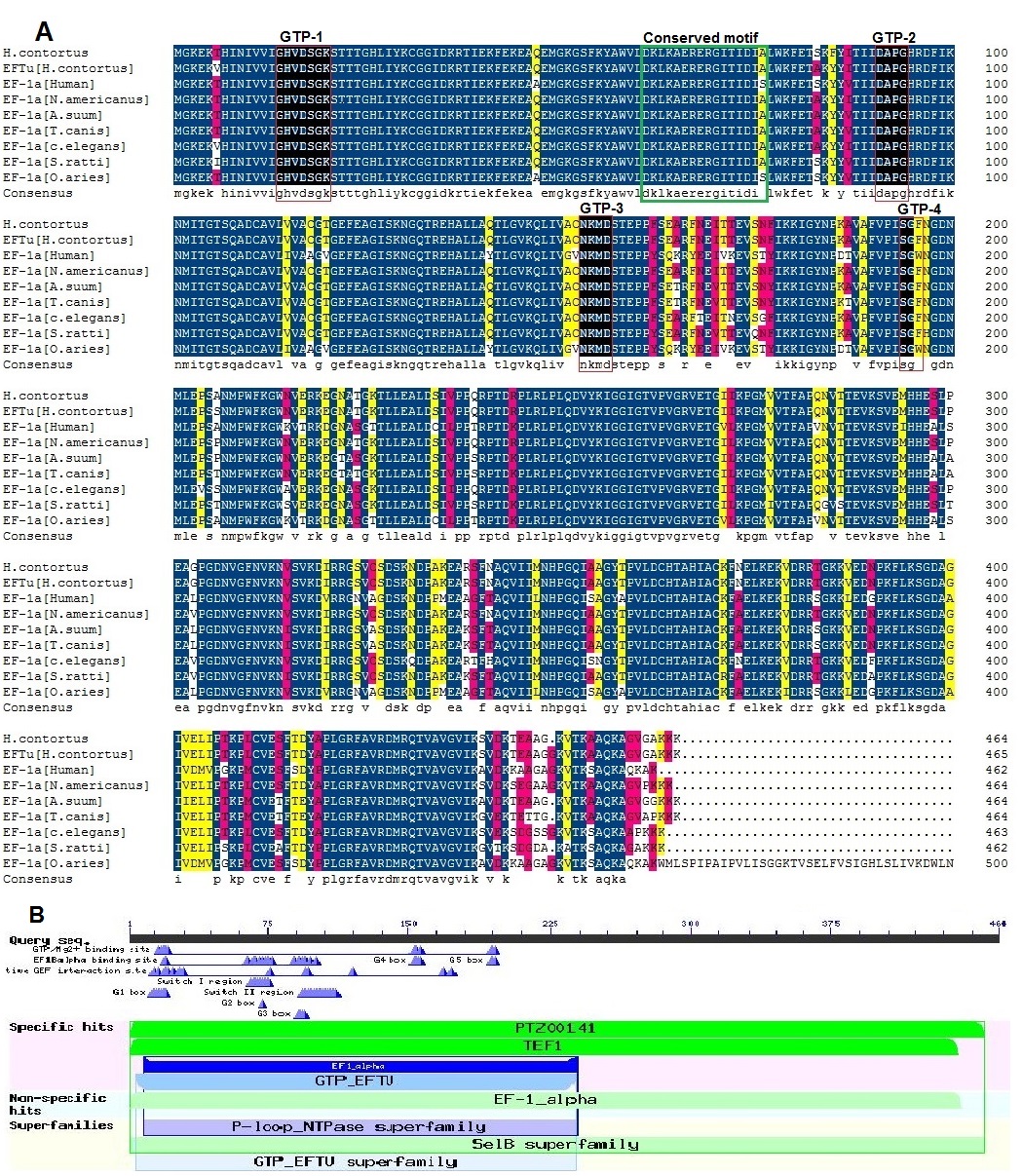


**Additional file 1:** Multiple sequence alignment and putative conserved domain of HcEF-1α amino acid sequence. A: NCBI database revealed that H. contortus EF-1α was closely related to the H. contortus (CDJ82784.1), Homo sapiens (AAH71841.1), Caenorhabditis elegans (NP_498520.1), Necator americanus (XP_013295799.1), Ascaris suum (DAA05869.1), Toxocara canis (KHN88882.1), Strongyloides ratti (CEF65912.1), Ovis aries (XP_014953624.1). The residues in white colour (blue back ground) are highly conserved among all species. The conserved domains for GTP binding sites (GTP-1, 2, 3 and 4) are boxed in red color and GTP-binding elongation factor signature sequence is boxed in green colour. B: The putative conserved domains related to HcEF-1α.
