# Supplementary material for: Recombinant elongation factor 1 alpha of Haemonchus contortus affects the functions of goat PBMCs
Source: Parasite Immunol. 2020 Feb 28;42(5):e12703. doi: 10.1111/pim.12703 (PMC7187238; doi:10.1111/pim.12703)
Supplement: Supplementary file 2 — Additional fileS2 [file PIM-42-e12703-s002.docx]

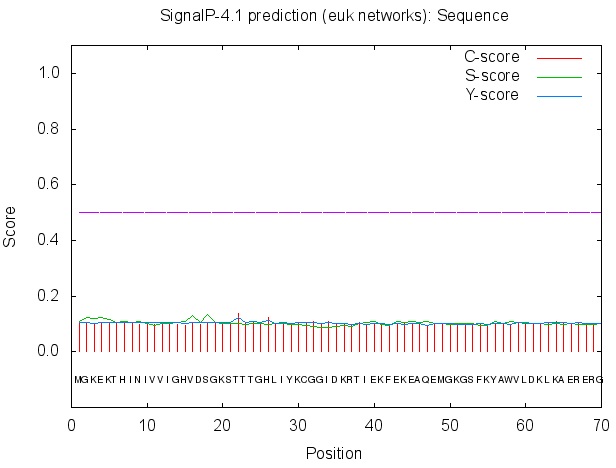


**Additional file 2:** N-terminal signal peptide prediction. The amino acid sequences of HcEF-1α (GenBank/Uniprot: HCOI_00777800/ U6NYV7) was used to predict N-terminal signal peptides by SignalP 4.1 Server.
