# Supplementary material for: Recombinant elongation factor 1 alpha of Haemonchus contortus affects the functions of goat PBMCs
Source: Parasite Immunol. 2020 Feb 28;42(5):e12703. doi: 10.1111/pim.12703 (PMC7187238; doi:10.1111/pim.12703)
Supplement: Supplementary file 3 — Additional fileS3 [file PIM-42-e12703-s003.docx]

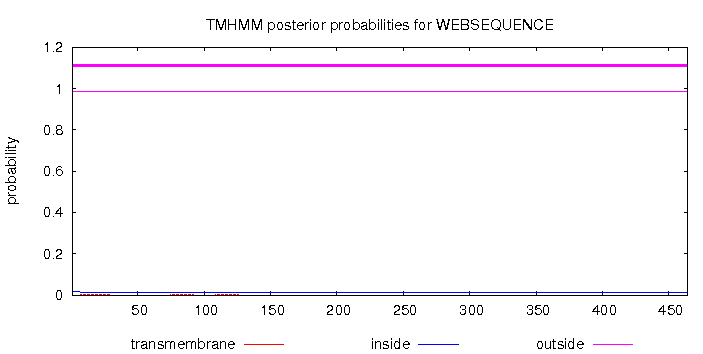


**Additional file 3:** Membrane protein prediction using TMHMM Server v.2.0. The amino acid sequences of HcEF-1α (GenBank/Uniprot: HCOI_00777800/ U6NYV7) was analysed to predict transmembrane structures using TMHMM Server v.2.0. There was no transmembrane domains was predicted in this protein structure. <http://www.cbs.dtu.dk/services/TMHMM/>.
