# Supplementary material for: Recombinant elongation factor 1 alpha of Haemonchus contortus affects the functions of goat PBMCs
Source: Parasite Immunol. 2020 Feb 28;42(5):e12703. doi: 10.1111/pim.12703 (PMC7187238; doi:10.1111/pim.12703)
Supplement: Supplementary file 4 — Additional fileS4 [file PIM-42-e12703-s004.docx]

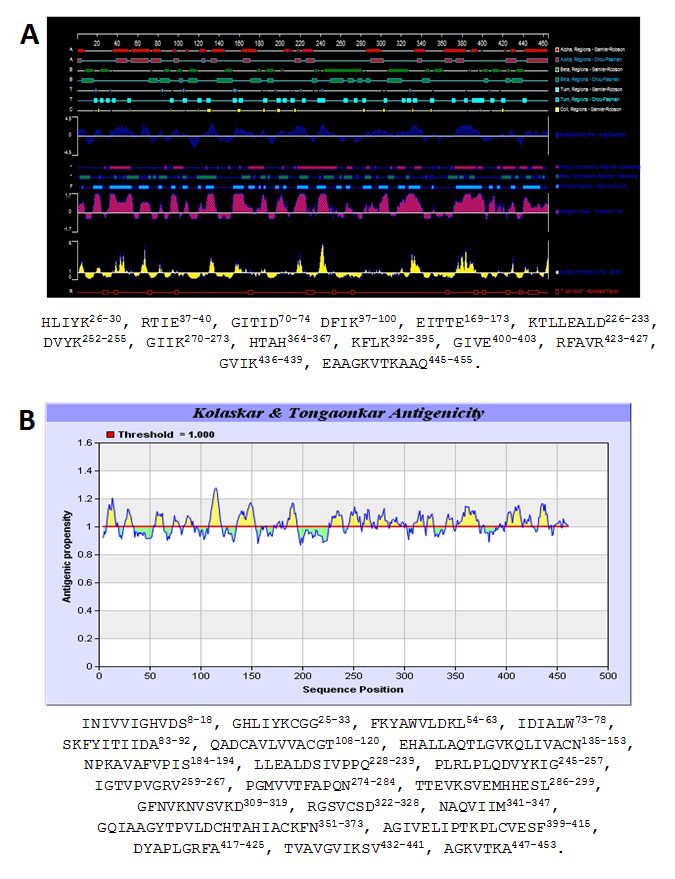


**Additional file 4:** Prediction of T and B cell epitopes for HcEF-1α: (A) Protein sequence of HcEF-1α (GenBank/Uniprot: HCOI_00777800/ U6NYV7) was used for the prediction of the T cell epitopes, that revealed 14 peptides of T cell epitopes. (B) Prediction of the B cell epitopes, which revealed 21 peptides of B cell epitopes.
